# NK cell receptor profiling of endometrial and decidual NK cells reveals pregnancy-induced adaptations

**DOI:** 10.3389/fimmu.2024.1353556

**Published:** 2024-03-20

**Authors:** Dorien Feyaerts, Marilen Benner, Gaia Comitini, Wijs Shadmanfar, Olivier W.H. van der Heijden, Irma Joosten, Renate G. van der Molen

**Affiliations:** ^1^ Department of Laboratory Medicine, Laboratory for Medical Immunology, Radboud University Medical Center, Nijmegen, Netherlands; ^2^ Mildred Clinics Arnhem, Arnhem, Netherlands; ^3^ Department of Obstetrics and Gynaecology, Radboud University Medical Center, Nijmegen, Netherlands

**Keywords:** natural killer cell, KIR, uterine, decidua, endometrium

## Abstract

Natural killer (NK) cells, with a unique NK cell receptor phenotype, are abundantly present in the non-pregnant (endometrium) and pregnant (decidua) humanuterine mucosa. It is hypothesized that NK cells in the endometrium are precursors for decidual NK cells present during pregnancy. Microenvironmental changes can alter the phenotype of NK cells, but it is unclear whether decidual NK cell precursors in the endometrium alter their NK cell receptor repertoire under the influence of pregnancy. To examine whether decidual NK cell precursors reveal phenotypic modifications upon pregnancy, we immunophenotyped the NK cell receptor repertoire of both endometrial and early-pregnancy decidual NK cells using flow cytometry. We showed that NK cells in pre-pregnancy endometrium have a different phenotypic composition compared to NK cells in early-pregnancy decidua. The frequency of killer-immunoglobulin-like receptor (KIR expressing NK cells, especially KIR2DS1, KIR2DL2L3S2, and KIR2DL2S2 was significantly lower in decidua, while the frequency of NK cells expressing activating receptors NKG2D, NKp30, NKp46, and CD244 was significantly higher compared to endometrium. Furthermore, co-expression patterns showed a lower frequency of NK cells co-expressing KIR3DL1S1 and KIR2DL2L3S2 in decidua. Our results provide new insights into the adaptations in NK cell receptor repertoire composition that NK cells in the uterine mucosa undergo upon pregnancy.

## Introduction

Uterine natural killer (NK) cells are one of the most abundant immune cells in both non-pregnant endometrium (1) and in early-pregnancy decidua (2). NK cell numbers in the endometrium increase over the course of the menstrual cycle, preparing the endometrium for embryo implantation (3). After fertilization, NK cell numbers further increase resulting in 70% of all lymphocytes in first trimester decidua being NK cells (2). Both endometrium and decidua-derived NK cells show CD56^bright^CD16^-^ phenotype for the majority of NK cells, lack cytotoxic activity, and secrete cytokines such as IFN-γ, IP-10, and VEGF (4–6). During the normal menstrual cycle, NK cells in the pre-pregnancy endometrium are suggested to play a role in mucosal integrity, endometrial angiogenesis, and endometrial shedding (7–9), while decidual NK cells have been shown to regulate trophoblast invasion and spiral artery remodeling (2). NK cell function is regulated by NK cell receptors (NKR) such as killer-immunoglobulin-like receptors (KIR), immunoglobulin-like transcripts (LILRB), C-type lectin heterodimer family (NKG2; e.g., NKG2A, NKG2C, NKG2D), and natural cytotoxicity receptors (NCR; e.g., NKp30, NKp44, NKp46) (10). The interaction between NKR on decidual NK cells and their respective ligands on trophoblast cells is suggested to play a role in controlling effective placentation (6, 11–13). Both endometrial and decidual NK cells have a unique NKR repertoire and are biased towards KIR2D expression (4, 14–16). It is speculated that tissue-resident NK cells in the endometrium contribute to the pool of decidual NK cells present during pregnancy, an observation confirmed in parabiosis studies in mice, where decidual NK cells developed from proliferating tissue-resident NK cells in the non-pregnant uterus (17). In addition, after human pregnancy, precursors for pregnancy-induced memory decidual NK cells were found to be present in the endometrium (18, 19). Microenvironmental changes can alter the phenotype of NK cells (20) and it has been suggested that pregnancy-specific factors can influence the receptor repertoire of decidual NK cells (14, 15, 21). However, it is still unclear to what extent the phenotype of decidual NK cells differs from its precursors in the endometrium. Previous studies have been limited to comparing endometrial or decidual NK cells with their peripheral counterparts (5, 14, 22), and consequently a direct and thorough assessment of the phenotypic differences and similarities of endometrial and decidual NK cells is lacking. To address whether phenotypic modifications of decidual NK cell precursors occurs under the influence of pregnancy, we evaluated the NKR repertoire of endometrial and early-pregnancy decidual NK cells.

## Materials and methods

### Tissue sampling

Menstrual blood was collected from 26 healthy women during the first 36 hours of menstruation, as described earlier (4, 23). Decidual tissue from 14 women undergoing elective termination of normal pregnancy between 6 and 15 weeks of gestation was collected at the Mildred Clinic Arnhem (Arnhem, the Netherlands). Tissues from elective terminations were donated anonymously. As such, demographics information such as age and parity/gravidity is not available for these samples. Use of menstrual blood and elective termination material was approved by the institutional review board (*Commissie Mensgebonden Onderzoek* region Arnhem-Nijmegen, CMO nr. 2009/004 and 2017-3253) and was performed in accordance with relevant guidelines and regulations. Samples were obtained from each participant upon written informed consent.

### Isolation of decidual lymphocytes

Decidual lymphocytes were isolated as previously described (24). Placental tissue was rinsed with PBS to remove blood clots. Trophoblast tissue was removed and decidual tissue was cut into small pieces. 5 ml of tissue was transferred to a C-tube and digested with 10 ml of Accutase (Thermo Fisher Scientific) for 60 minutes in a 37°C water bath while shaking. Tissue was processed twice with a gentleMACS dissociator (program h-tumor 02; Miltenyi Biotech) before and after 30 minutes of digestion. The obtained supernatant was passed through a 100 µm, 70 µm, and 40 µm cell strainer to obtain a single cell suspension. After washing of the collected cell suspension with supplemented RPMI [RPMI 1640 medium supplemented with pyruvate (1 mM), glutamax (2 mM), penicillin (100 U/ml), streptomycin (100 mg/ml); Thermo Fisher Scientific], cells were layered on a discontinuous Percoll gradient (1,050 g/ml, 1,056 g/ml, and 1,084 g/ml; GE Healthcare) for density gradient centrifugation (801 x g for 15 minutes, no brake). Lymphocytes were isolated from the 1,084-1,056 g/ml interface and washed twice with supplemented RPMI before further processing.

### Isolation of endometrial lymphocytes

Endometrial immune cells were isolated from menstrual blood as described before (4, 23). In brief, menstrual blood was washed with PBS and passed through a 70 µm cell strainer to remove mucus and blood clots. Granulocytes were depleted by using the human granulocyte depletion cocktail RosetteSep™ according to manufacturer’s protocol (Stemcell technologies Inc). After Ficoll density gradient centrifugation (Lymphoprep), menstrual blood mononuclear cells were collected and washed twice with 2%HPS-PBS (HPS, human pooled serum, manufactured in-house) before further processing.

### Flow cytometry of decidual and endometrial lymphocytes

Cells were washed twice with 0.2% bovine serum albumin-PBS (BSA, 0.2% v/v; Sigma-Aldrich), followed by staining for 20 min at room temperature in the dark. After washing twice with 0.2% BSA-PBS, cells were analyzed using 10-color staining panels on the 10-color flow cytometer Navios™ (480 nm argon blue laser, 405 nm solid state violet laser, 636 nm solid state laser, Beckman Coulter). Data analysis was performed using Kaluza 2.1 software (Beckman Coulter).

The following fluorochrome-conjugated antibodies were used to phenotypically characterize NK cells in decidua and endometrium: CD3-APC-AF750, CD16-FITC, CD45-KO, CD56-ECD (Beckman Coulter), CD85j-PC5.5 (LILRB1; Beckman Coulter, custom made), CD158a-AF700 (KIR2DL1; R&D systems), CD158a/h-PC5.5/APC-AF700 (KIR2DL1/S1; Beckman Coulter, custom made), CD158b2-FITC (KIR2DL3; R&D systems), CD158b1/b2-PC7 (KIR2DL2/L3/S2; Beckman Coulter), CD158e1-BV421 (KIR3DL1; BioLegend), CD158e1/e2-APC (KIR3DL1/S1; Beckman Coulter), CD159a-APC/PB (NKG2A; Beckman Coulter, custom made), CD159c-PE (NKG2C; R&D systems), CD161-PB, CD244-APC-AF700 (2B4; Beckman Coulter, custom made), CD314-APC (NKG2D), CD335-PC7 (NKp46), CD336-PE (NKp44; Beckman Coulter), and CD337-PC5.5 (NKp30; Beckman Coulter, custom made). The gating strategy used for analysis of NK cells in decidua and menstrual blood is depicted in **Supplementary Figure S1**. As depicted in **Supplementary Figure S1**, gates were decided based on comparing marker expression on CD56^-^ and CD56^+^ cells. KIR co-expression patterns were calculated using the "Tree" function in Kaluza software, which looks for co-expression patterns between markers for which single gates have been identified. KIR co-expression patterns were obtained similarly to our previous manuscript (4). This co-expression pattern focused on receptors that can recognize HLA-C, HLA-G, and HLA-E on trophoblast cells. This combination of receptors was only present in one 10-color flow cytometry panel. Moreover, the co-expression of other receptors on NKG2a^+/-^ cells, such as NKp30, are not depicted here since these markers are not included in the same staining panel as NKG2a.

### Statistical analysis

Non-parametric Mann-Whitney test was used to compare decidual and endometrial NK cells. Simple linear regression analysis was used to analyze NKR expression changes over gestation. Statistical analyses were performed using GraphPad Prism 9 and p-values < 0.05 were considered significant. All values are shown as mean.

## Results

### NK cell composition of decidua differs from endometrium

**Figure 1:**
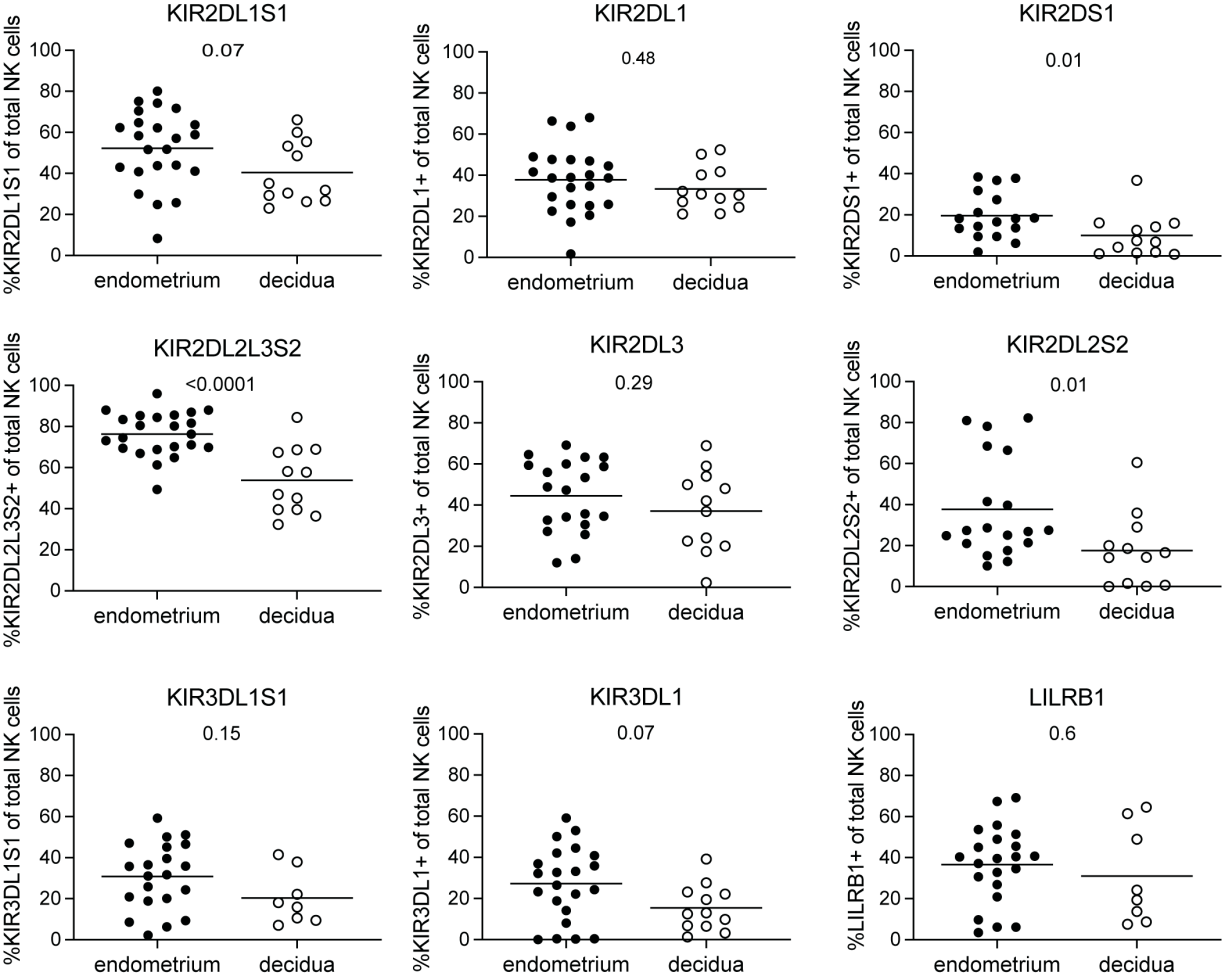
KIR and LILRB1 receptor expressing NK cells in endometrium and decidua. Percentages of NK cells expressing KIR2DL1S1, KIR2DL1, KIR2DS1, KIR2DL2L3S2, KIR2DL3, KIR2DL2S2, KIR3DL1S1, KIR3DL1, and LILRB1 for NK cells in endometrium and decidua. P-values are indicated on the graphs.

**Figure 2:**
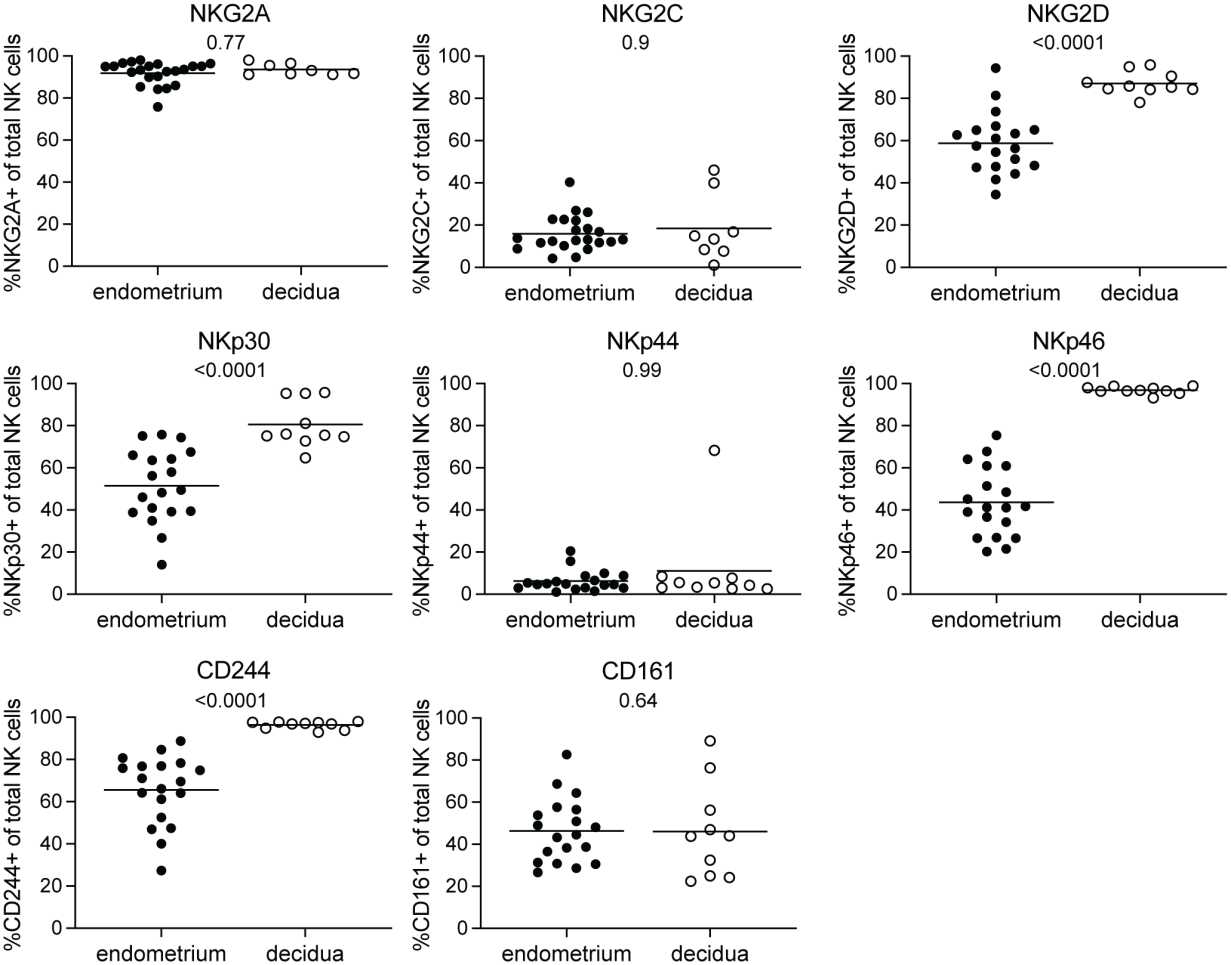
NKG2 and NCR receptor expressing NK cells in endometrium and decidua. Percentages of NK cells expressing NKG2A, NKG2C, NKG2D, NKp30, NKp44, NKp46, CD244, and CD161 for NK cells in endometrium and decidua. P-values are indicated on the graphs.

At the maternal-fetal interface, decidual NK cells are involved in controlling correct placentation during the first trimester of pregnancy (2). NK cells in the non-pregnant endometrium have been implied to be precursors of decidual NK cells (17, 18). It is unclear how the phenotype of decidual NK cells in early pregnancy compares to that of their endometrial precursors. To this end, we collected pre-pregnancy endometrial NK cells derived via menstrual blood and compared their NKR immunophenotype to decidual NK cells obtained from placental tissue of elective pregnancy terminations between 6 and 15 weeks of gestation. To characterize the NKR repertoire, we evaluated the expression of 17 NKRs suggested to play a role at the maternal-fetal interface using flow cytometry (6, 11–13, 21). Our results showed that KIR2DS1, KIR2DL2L3S2, and KIR2DL2S2 expressing NK cells were present at significantly lower frequencies in decidua compared to endometrium (**Figure 1**). No significant differences were observed for NK cells expressing LILRB1, KIR2DL1S1, KIR2DL1, KIR2DL3, KIR3DL1, and KIR3DL1S1. Moreover, KIR2DS1 expressing NK cells decreased significantly over the course of pregnancy in the decidua (**Supplementary Figure S2**). Next to KIR receptors, it has been shown that endometrial and decidual NK cell also express NCR and other NKR (5, 18, 21, 25). We observed that frequencies of NK cells in the decidua expressing NKG2D, NKp30, NKp46, and CD244 were significantly higher compared to NK cells in endometrium (**Figure 2**). We did not find a significant difference for NKG2A, NKG2C, NKp44 or CD161 expressing NK cells. Frequencies of NKG2 and NCR expressing NK cells in the decidua did not change over the course of gestation (**Supplementary Figure S3**). Overall, this clearly showed that the phenotype and cell composition of early pregnancy NK cells in the decidua differ from their precursors of in the endometrium, with less KIR^+^ NK cells and more NKG2^+^ and NCR^+^ cells in decidua compared to endometrium.

### Decidual NK cells have a distinct co-expression pattern compared to endometrial NK cells

**Figure 3:**
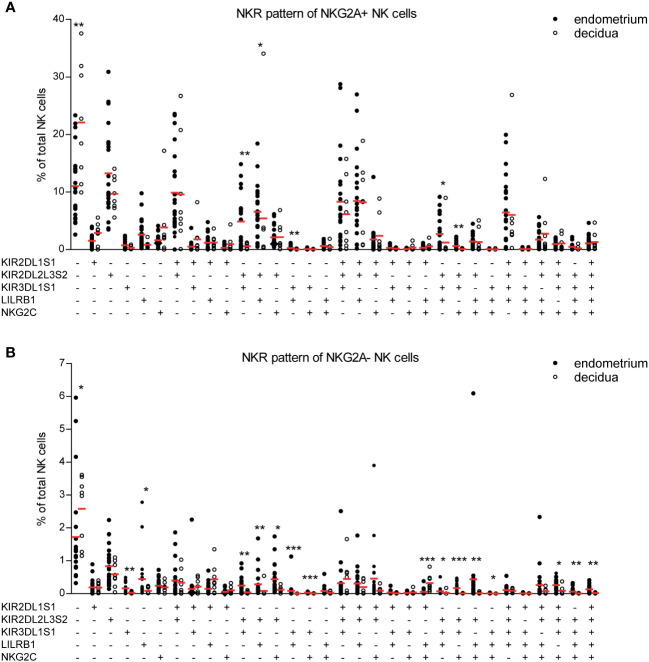
NK cell receptor co-expression pattern is different for NK cells derived from endometrium and decidua. **(A, B)** Percentage of each NK cell subpopulation (KIR2DL1S1+/-KIR2DL2L3S2+/-KIR3DL1S1+/-LILRB1+/-NKG2C+/-NKG2A+/-) is shown as a percentage of total NK cells in endometrium and decidua. **(A)** NKG2A+ and **(B)** NKG2A- subpopulations are shown separately. *P < 0.05, **P < 0.01, ***P < 0.001.

Next, we sought to asses co-expression patterns on decidual and endometrial NK cells as NK cell function is tightly controlled by the co-expression of a variety of activating and inhibitory receptors (26). This receptor co-expression results in a high degree of NK cell diversity, with an estimate of 6,000 to 30,000 different phenotypic NK cell subpopulations (27). Microenvironment changes such as viral infections can skew this NK cell subpopulation composition (28). We and others have shown that both endometrium and decidua contain a unique profile of NK cell subpopulations compared to peripheral blood (4, 16, 26). To address whether pregnancy induces a change in NK cell subpopulation composition in the uterine mucosa, we identified 64 distinct NK cell subpopulations based on the co-expression of receptors that can recognize HLA-C, HLA-G, and HLA-E on trophoblast cells, i.e., KIR2DL1S1, KIR2DL2L3S2, KIR3DL1S1, LILRB1, NKG2A, and NKG2C. **Figures 3A, B** were separated based on NKG2a expression, as the majority of uterine NK cells are NKG2A^+^. Combining both NKG2A^+^ and NKG2A^-^ in the same figure would make NKG2A^-^ cells not as visible on the y-axis due to scaling differences. We observed that different NK cell subpopulations were present in decidua versus endometrium (**Figures 3A, B**). For example, while almost absent in decidual tissue, the endometrial NK cell population clearly contained KIR2DL2L3S2^+^KIR3DL1S1^+^, KIR2DL2L3S2^+^ KIR3DL1S1^+^LILRB1^+^, and KIR2DL2L3S2^+^KIR3DL1S1^+^NKG2C^+^ NK cells. The majority of NK cell subpopulations that showed significant differences between the decidua and endometrium co-expressed KIR3DL1S1 and KIR2DL2L3S2, along with other NK cell receptors. These subpopulations were found at lower frequencies in decidual NK cell populations. This suggests that upon pregnancy, the uterine NK cell population changes, including a decrease in NK cells co-expressing KIR3DL1S1 and KIR2DL2L3S2. In line with previous results (4), we showed a high frequency of NK cells co-expressing three or more HLA-recognizing receptors in endometrium and decidua (**Supplementary Figure S4**). In addition, significantly more decidual NK cells expressed none of the measured NKR, or only 1 NKR, compared to endometrium, which, in accordance with Sharkey et al. (26), is mainly due to a high frequency of NKG2A^+^ decidual NK cells.

## Discussion

NK cells are abundantly present in the uterine mucosa (1, 2), where they support important processes in endometrial mucosal integrity and placentation (2, 7–9). It is hypothesized that NK cells in non-pregnant endometrium are precursors of decidual NK cells present during pregnancy (17, 18). To assess whether phenotypic modifications of these NK cell precursors occur under influence of pregnancy, we compared the NK cell receptor repertoire of endometrial and early-pregnancy decidual NK cells using 10-color flow cytometry. We showed that NK cells in pre-pregnancy endometrium have a different phenotype and cell composition compared to NK cells in the pregnant decidua. This suggests that the microenvironment during pregnancy can change the phenotypic composition of decidual NK cell precursors.

We showed that the frequency of KIR2DS1, KIR2DL2L3S2, and KIR2DL2S2 expressing NK cells was significantly lower in decidua, together with decreasing frequency of KIR2DS1^+^ decidual NK cells with increasing gestational age, which is in line with previous studies showing that the phenotype of first trimester decidual NK cells is dynamic and marked by a decrease of KIR2DL1S1 and KIR2DL2L3S2 expressing decidual NK cells throughout the first trimester (15, 21). This decrease in receptor expression might be due to increased interactions of these receptors with their respective MHC-ligands on trophoblast cells (14, 29, 30). However, previous studies showed that fetal HLA-C genotype did not affect KIR expression on decidual NK cells (14, 26). Instead, the frequency of KIR-expressing decidual NK cells was influenced by the maternal HLA-C genotype (14, 26). Stromal cells, which are in close contact with decidual NK cells at the maternal-fetal interface, are also known to express KIR ligands, and their expression increases after fertilization (14, 30). This might imply that stimulation through ligands on maternal-derived stromal cells is more likely to change the KIR receptor expression of decidual NK cells than stimulation through trophoblast cells (14). In addition to ligand binding, also chemokines or cytokines secreted by trophoblast cells and/or stromal cells could potentially account for the changes observed. For instance, culturing of peripheral NK cells with TGFβ1 or conditioned medium derived from decidual stromal cells resulted in accumulation of NK cells with a decidual NK cell phenotype, i.e. increased CD56 intensity, loss of CD16, and increased frequency of KIR^+^ NK cells (31). Moreover, IL-15 has been suggested to drive uterine NK cell differentiation (32). These phenotypic composition changes could have an impact on the functional capacity of decidual NK cells. For example, activating KIR2DS1^+^ decidual NK cells by HLA-C2 binding increased the production of GM-CSF, which enhanced the migration of trophoblast cells (12). As such, decreasing the presence of KIR2DS1^+^ NK cells might limit excessive activation of decidual NK cells and too extensive trophoblast invasion.

Next to a decrease in KIR-expressing NK cells, we showed that the frequency of NK cells expressing activating receptors NKG2D, NKp30, NKp46, and CD244 significantly higher in the decidua compared to endometrium. This higher receptor expression could be mediated by increasing IL-15 levels upon fertilization (5), as IL-15 stimulation has been shown to enhance the expression of these receptors on NK cells (5, 33). Stimulation of these receptors on decidual NK cells results in increased production of chemokines, cytokines, and angiogenic factors, which, in turn, have been shown to promote vascular growth and trophoblast invasion (6, 11, 34). Therefore, this increase in NK cells expressing these activating receptors, together with the decrease in KIR^+^ NK cells, could have an impact on the functional capacity of decidual NK cells and might be necessary for controlling placentation. Moreover, MICA and MICB, ligands for NKG2D, are induced upon cellular stress during pathogenic infection (35) and NKG2D on decidual NK cells is involved in cytotoxic killing of virus-infected decidual fibroblasts (36). Therefore, increased NKG2D expression may help protect the fetus from intrauterine viral infections.

NKR co-expression patterns showed a lower frequency of NK cells co-expressing KIR3DL1S1 and KIR2DL2L3S2 in the decidua compared to endometrium. Few studies have examined NK cell subpopulations in human tissues, and it remains poorly understood which factors determine the decrease or increase of certain NK cell subpopulations. We and others speculate that both ligand stimulation and soluble factors could contribute to the observed changes (14, 26, 29, 30). Future research should aim to determine the contribution of these individual NK cell subpopulations to their role in the uterine mucosa.

Recent single-cell RNA sequencing and cytometry studies have revealed three distinct uterine NK cell subpopulation: uNK1, uNK2, and uNK3 (37–39). These studies showed that the majority of first trimester uNK cells are uNK1 cells and that the composition of these uterine NK cell subsets changes compared to non-pregnant endometrium and across gestation. Moreover, Huhn et al. (39) showed that a small fraction of CD56dimCD16^+^ conventional NK cells are present in first trimester decidua. Unfortunately, we did not acquire these specific markers (CD57, CD49a, Eomes, Tbet, CD69, CD103) in our staining panels to distinguish these populations and as such we cannot assess NKR repertoire differences within these subsets.

While we used menstrual blood as a proxy for endometrial immunity, significant differences have been observed between secretory, proliferative, and menstrual phase NK cell immunity. NK cell abundance increases over the course of the menstrual cycle (40). While differences in marker expression can be observed between biopsy and menstrual blood-derived endometrial NK cells (32, 41), it is important to note that very few studies compared endometrial biopsy and menstrual blood head-to-head. In addition, also a lot of similarities can be observed. For instance, in a recent study by Whettlock et al. (38) the frequency of uNK1, uNK2, and uNK3 subsets did not significantly differ between menstrual, proliferative, and secretory phase, and also KIR2DL1, KIR2DL2L3, and LILRB1^+^ NK cell frequencies did not differ significantly over the menstrual cycle. As such, while menstrual blood seems to be a good substitute for investigation endometrial immunity, a thorough assessment of NK cell subsets in biopsy- vs. menstrual blood-derived endometrium, preferably in the same individual, is still needed.

It has become clear that the NKR repertoire of decidual NK cells during pregnancy is different compared to the non-pregnant situation, which may have important consequences for correct placentation and protection during intrauterine infection. It can be envisaged that complications such as implantation failure or miscarriage could result from dysregulated changes in NKR expression. Several reports have highlighted a different NK cell phenotype on decidual NK cells from complicated pregnancies compared to non-complicated pregnancies (42–45). Therefore, investigating whether decidual NK cell precursors in the endometrium of women with pregnancy complications fail to adapt into decidual NK cells could improve our understanding of the underlying pathogenesis and may have future utility in providing therapeutic targets. It will be of interest to understand which factors, either through ligand stimulation or soluble factors, contribute to the phenotypic changes observed here.

Our study has several limitations. Firstly, we had no access to paired samples, i.e. endometrial and decidual NK cells from the same individual. Paired samples would shed light onto the specific changes NK cells undergo in a single individual. However, this poses inconceivable logistic and ethical barriers. Secondly, the limited size of decidua tissue samples here poses a constraint for a comprehensive analysis of the dynamic changes in NKR expression composition across gestation. Unfortunately, we lack KIR genotyping results for the donors of the abortion material, preventing us from making adjustments to the expression data for those specific samples. Lastly, while enzymatic digestion of tissue can affect surface protein expression, we and others (32) did not observe an effect on NKR markers.

In conclusion, in this study we have demonstrated that decidual NK cell precursors change their NK cell receptor repertoire composition upon pregnancy. We showed that the phenotype and cell composition of pre-pregnancy endometrial NK cells differs significantly from decidual NK cells. The frequency of several KIR^+^ NK cells was lower upon pregnancy, while the frequency of NK cells with activating receptors such as NKp30, NKp46, and NKG2D was higher. In addition, less NK cells co-expressing KIR3DL1S1 and KIR2DL2L3S2 were present in the decidual NK cell population. This suggests that the uterine environment, either through secretion of soluble factors or by ligand stimulation, changes the phenotype of uterine NK cells upon pregnancy. This change in receptor expression may be important in controlling placentation. Future research should be aimed at investigating the factors involved in these phenotypic changes and the role of different NK cell subpopulations in pregnancy success.

## Data availability statement

The raw data supporting the conclusions of this article will be made available by the authors, without undue reservation.

## Ethics statement

The studies involving humans were approved by Commissie Mensgebonden Onderzoek region Arnhem-Nijmegen. The studies were conducted in accordance with the local legislation and institutional requirements. The participants provided their written informed consent to participate in this study.

## Author contributions

DF: Conceptualization, Data curation, Formal Analysis, Investigation, Methodology, Visualization, Writing – original draft, Writing – review & editing. MB: Investigation, Methodology, Writing – review & editing. GC: Investigation, Methodology, Writing – review & editing. WS: Writing – review & editing. Ov: Writing – review & editing. IJ: Conceptualization, Supervision, Writing – review & editing. RV: Conceptualization, Funding acquisition, Resources, Supervision, Writing – original draft, Writing – review & editing.

## Supplementary material

Supplementary Figure 1 Gating strategy and FACS plots of NK cell receptor expression. Representative staining for NK cell receptors on NK cells in **(A)** endometrium and **(B)** decidua.

Supplementary Figure 2 KIR and LILRB1 receptor expressing NK cell frequencies change over the course of pregnancy. Percentages of NK cells expressing KIR2DL1S1, KIR2DL1, KIR2DS1, KIR2DL2L3S2, KIR2DL3, KIR2DL2S2, KIR3DL1S1, KIR3DL1, and LILRB1 for NK cells in decidua, plotted according to gestational age. R^2^ values and p-values are indicated on the graphs.

Supplementary Figure 3 NKG2 and NCR receptor expressing NK cell frequencies change over the course of pregnancy. Percentages of NK cells expressing NKG2A, NKG2C, NKG2D, NKp30, NKp44, NKp46, CD244, and CD161 for NK cells in decidua, plotted according to gestational age. R^2^ values and p-values are indicated on the graphs.

Supplementary Figure 4 NK cell receptor (NKR) co-expression pattern. Cumulative percentage of NK cells expressing 0, 1, 2, 3, 4, 5, or six HLA-recognizing NKR (mean ± SD). Percentages calculated based on receptor expression corresponding to **Figure 3**. **P* < 0.05.
