# Supplementary figures and images for: NK cell receptor profiling of endometrial and decidual NK cells reveals pregnancy-induced adaptations
Source: Front Immunol. 2024 Mar 20;15:1353556. doi: 10.3389/fimmu.2024.1353556 (PMC10987737; doi:10.3389/fimmu.2024.1353556)

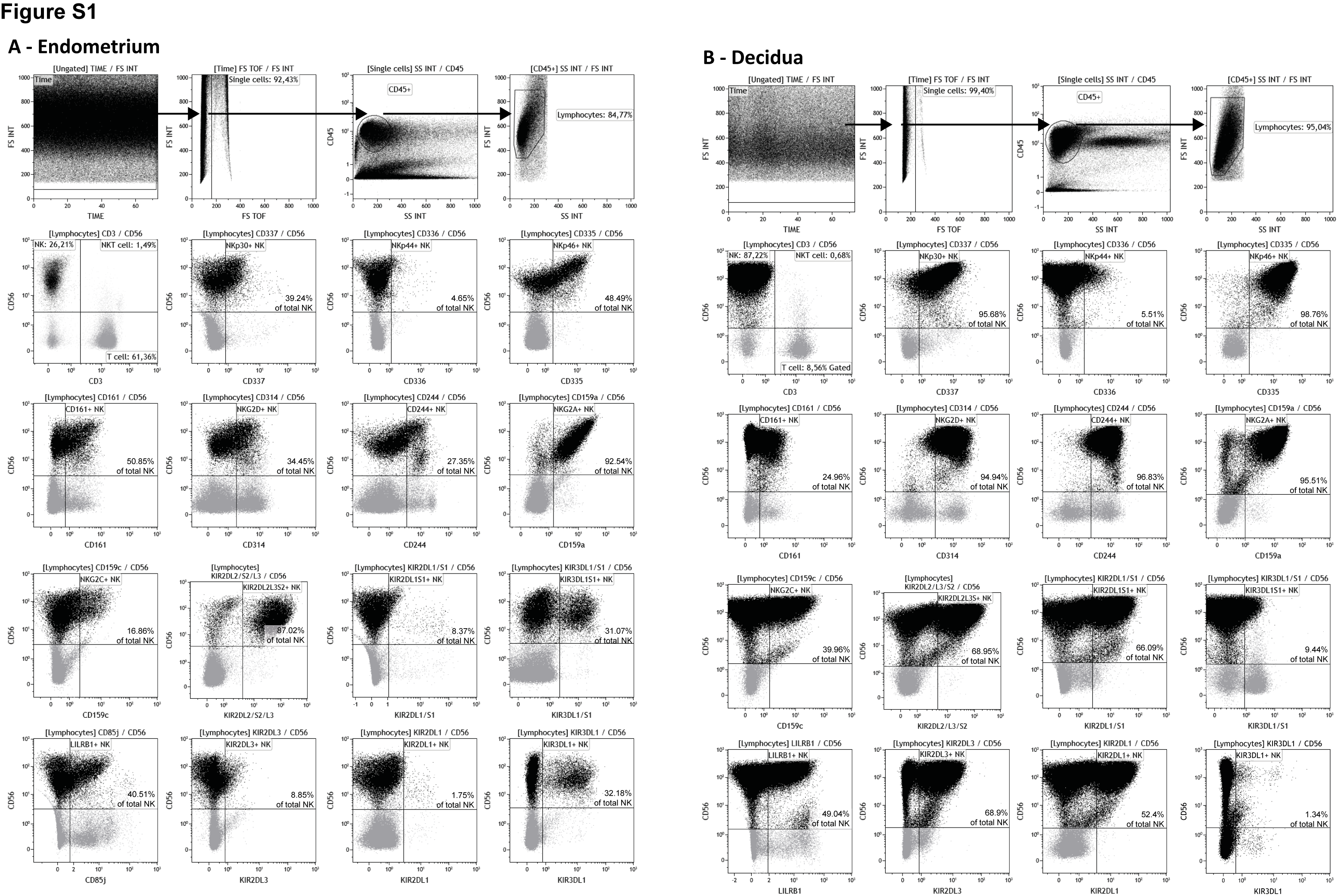

Supplement: Supplementary Figure 1 — Gating strategy and FACS plots of NK cell receptor expression. Representative staining for NK cell receptors on NK cells in (A) endometrium and (B) decidua. [file Image_1.tif]

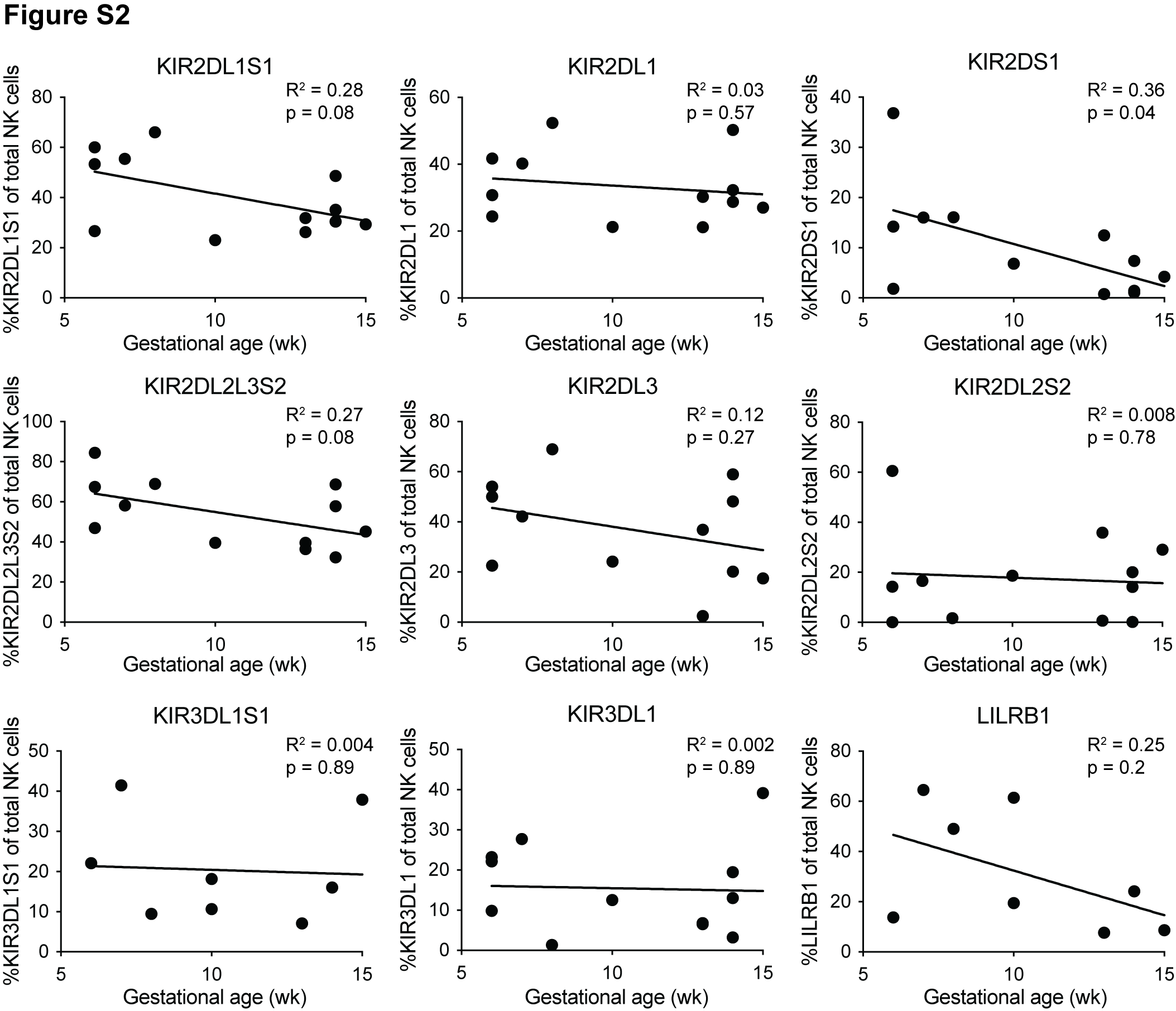

Supplement: Supplementary Figure 2 — KIR and LILRB1 receptor expressing NK cell frequencies change over the course of pregnancy. Percentages of NK cells expressing KIR2DL1S1, KIR2DL1, KIR2DS1, KIR2DL2L3S2, KIR2DL3, KIR2DL2S2, KIR3DL1S1, KIR3DL1, and LILRB1 for NK cells in decidua, plotted according to gestational age. R2 values and p-values are indicated on the graphs. [file Image_2.tif]

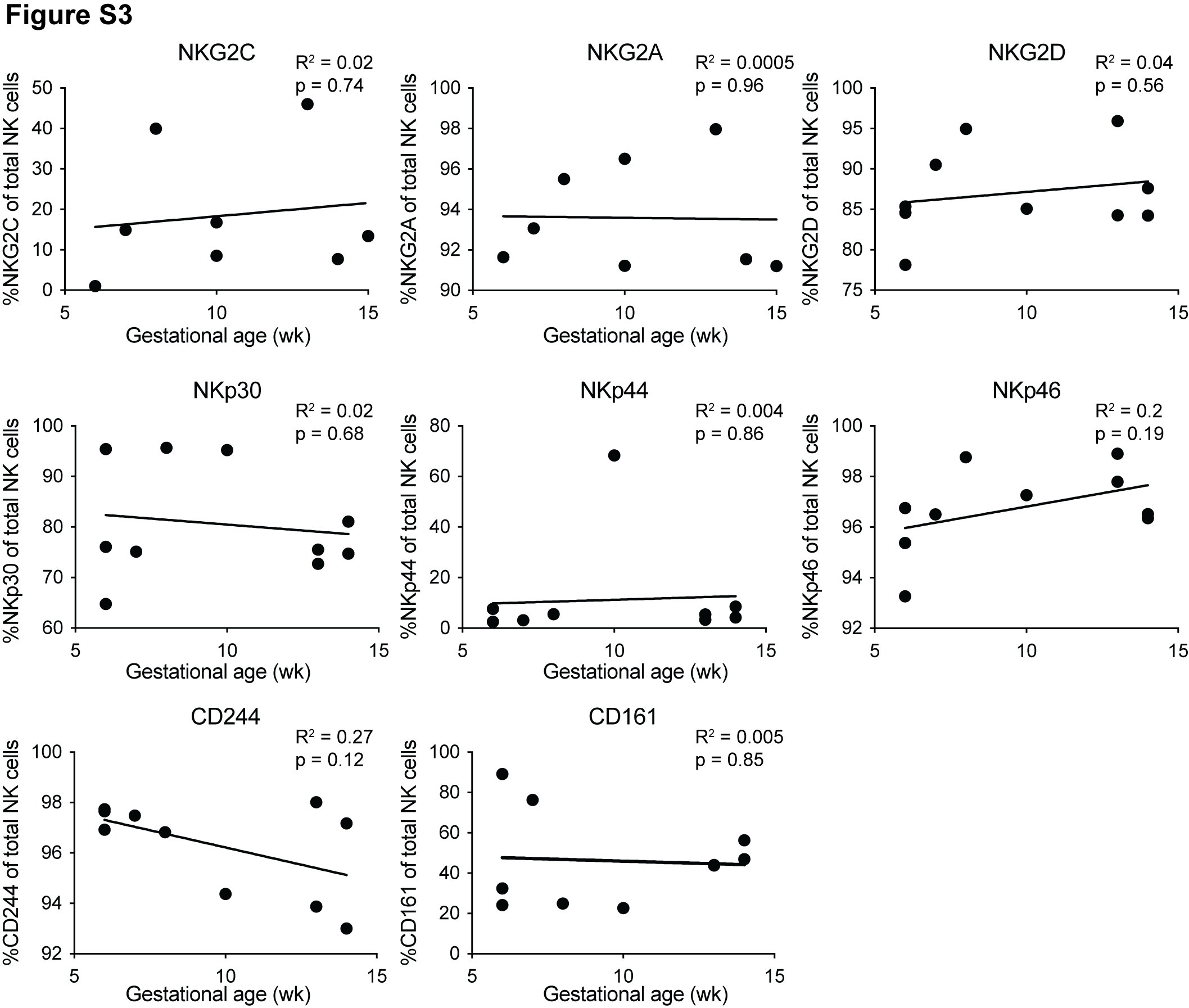

Supplement: Supplementary Figure 3 — NKG2 and NCR receptor expressing NK cell frequencies change over the course of pregnancy. Percentages of NK cells expressing NKG2A, NKG2C, NKG2D, NKp30, NKp44, NKp46, CD244, and CD161 for NK cells in decidua, plotted according to gestational age. R2 values and p-values are indicated on the graphs. [file Image_3.tif]

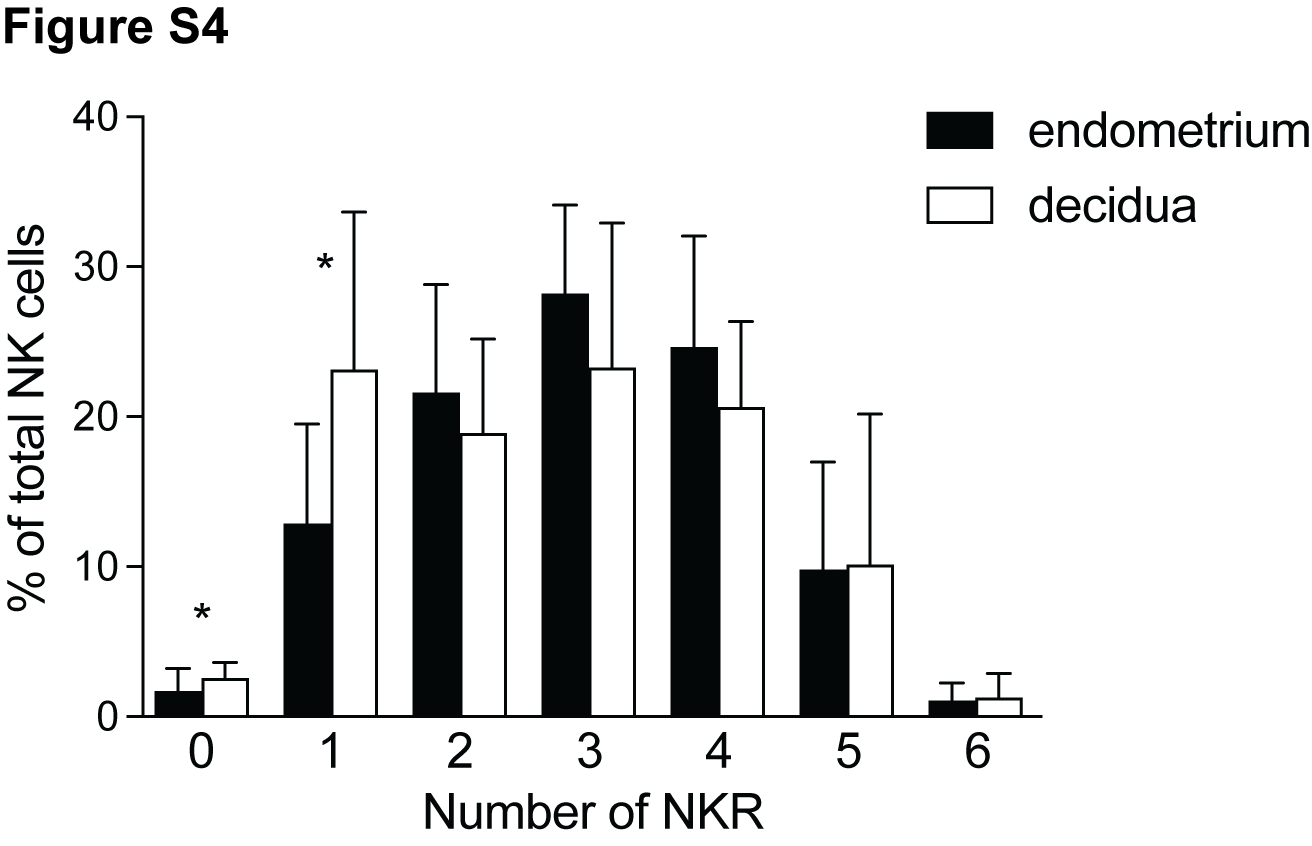

Supplement: Supplementary Figure 4 — NK cell receptor (NKR) co-expression pattern. Cumulative percentage of NK cells expressing 0, 1, 2, 3, 4, 5, or six HLA-recognizing NKR (mean ± SD). Percentages calculated based on receptor expression corresponding to Figure 3 . *P < 0.05. [file Image_4.tif]
